# Are hospitalized patients with inflammatory bowel disease at increased risk of invasive bacterial infections? Results from POLIBD 3-year cohort study

**DOI:** 10.1186/s13099-021-00408-6

**Published:** 2021-02-22

**Authors:** Jolanta Gruszecka, Rafał Filip

**Affiliations:** 1 Medical College of Rzeszow University Institute of Health Sciences, Rzeszow, Poland; 2 Department of Clinical Microbiology, Clinical Hospital No. 2 im. Św. Jadwigi Królowej, Rzeszow, Poland; 3 Faculty of Medicine, University of Rzeszow, Rzeszow, Poland; 4 Department of Gastroenterology with IBD Unit of Clinical Hospital No 2 im. Św. Jadwigi Królowej, Rzeszow, Poland

**Keywords:** Inflammatory bowel disease, Bacteremia, Risk factors

## Abstract

The aim of this study was to determine the dominant species of bacteria found in blood cultures collected from patients under treatment in the tertiary inflammatory bowel disease (IBD) center in Poland. The dominant pathogen isolated from blood in patients with IBD was *Staphylococcus epidermidis* MRCNS (MRCNS—methicillin-resistant coagulase-negative *Staphylococcus*), a strain resistant to all beta-lactam antibiotics (penicillins, penicillins with B-lactamase inhibitor, cephalosporins and carbapenems). The second most commonly isolated pathogen found in the blood samples was *Escherichia coli*. Blood cultures were found to be positive for these pathogens more frequently in male patients (90.0%). An increased risk of bacteremia in IBD patients was associated with prolonged hospitalization.

## Main text

Patients with ulcerative colitis (UC) and Crohn’s disease (CD) have significantly increased risk of infections that are primarily a consequence of increased intestinal permeability caused by mucosal inflammation [1]. Altered gut barrier functioning may cause infections both within and outside of the gastrointestinal tract (GI), including the development of sepsis [2]. However, there are other factors that negatively influence overall immunity, adding to the increased risk of both viral and bacterial infections such as reduced nutritional status, and, most importantly, the wide use of immunomodulators (thiopurines, methotrexate and calcineurin inhibitors) alone or in combination with a variety of biological agents such as anti-TNFs, anti-integrins, inti-interleukin 12/23p40 or JAK inhibitors [3]. Specific reports on risk factors for bacteremia in the general population of hospitalized patients with IBD are limited to single reports and none of these pertain to Central Europe [4]. Therefore, the aim of this study was to assess bacteremia rates, the risk factors associated with bacteremia, and associated mortality rates among IBD patients hospitalized due to disease exacerbation.

The results of tests of adult patients admitted and treated from January 2017 to December 2019 at the tertiary IBD center in Rzeszow (southern Poland) were analyzed. The data for all hospitalized patients used for the analysis were taken from the hospital's electronic medical records. Patients with IBD were selected based on the international classification of Crohn's disease (CD) or ulcerative colitis (UC). Blood was drawn for culture when the patient had a temperature of 37 °C or higher. Bacteremia was diagnosed in the case of positive blood culture results with the presence of characteristic clinical symptoms. A minimum of 2 blood samples were collected from independent, anatomically separated puncture sites (right hand, left hand) immediately before starting empirical therapy. Blood collected from patients was placed in vials with media (room temperature) that allowed for aerobic or anaerobic culture and detection of microbial growth. Immediately after collection, the blood sample vials were transferred to the microbiology laboratory located within the hospital and incubated in a BACTEC apparatus (Becton Dickinson) for 7 days; incubation time was less if microbial growth was obtained prior to 7 days [5]. Positive samples were plated on Columbia Agar with 5% Sheep Blood and MacConkey Agar with Crystal Violet (from aerobic culture) or Columbia Agar with 5% Sheep Blood, MacConkey Agar with Crystal Violet and Schaedler Agar with 5% Sheep Blood (from anaerobic culture). Plates with Columbia Agar with 5% sheep blood and MacConkey Agar with crystal violet were incubated for 24 h at 37 °C. Plates with Schaedler Agar with 5% sheep blood were incubated for 48 h at 37 °C. Identification of the cultured microorganisms was carried out by mass spectroscopy (MALDI-TOF MS) using an automatic mass spectrometer VITEK MS (bioMérieux, France). The drug resistance profile of cultured and identified microorganisms was determined by the disc diffusion method, or means of a VITEK2 (bioMérieux, France) automatic system for identification and determination of susceptibility according to EUCAST (European Committee on Antimicrobial Susceptibility Testing) [6].

**Fig. 1:**
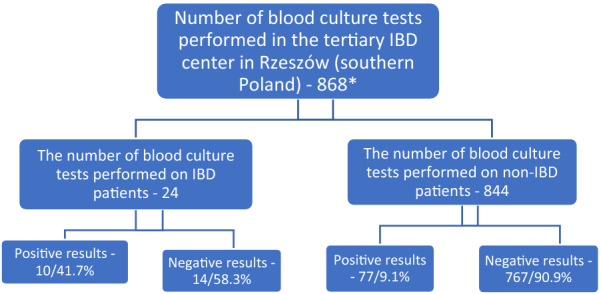
Percentages of positive and negative cultures in the population sampled. * The total number of patients treated in the tertiary IBD center in Rzeszow (southern Poland) during the study period was 2667

During the study period, 2667 adult patients were hospitalized in the tertiary IBD center in Rzeszow (southern Poland). A total of 868 blood culture tests were performed. Positive results were obtained in 87 trials. Bacteremia was found in 9.1% of non-IBD patients (77/844) and 41.7% of IBD patients (10/24)—Fig. 1.

An indication for hospitalization of IBD patients was disease exacerbation. The mortality rate after 30 days in patients with bacteremia was 0% (no deaths were observed in patients with IBD who were simultaneously diagnosed with bacteremia). Longer hospitalization times were associated with an increased risk of bacteremia. The most commonly diagnosed pathogens among IBD patients were coagulase-negative *Staphylococcus epidermidis* MRCNS (4/10 patients) and *Escherichia coli* (3/10 patients). In other cases, *Staphylococcus aureus* (1/10 patients), *Cutibacterium acnes* (gram-positive rod-shaped anaerobic bacteria) (1/10 patients) and *Micrococcus luteus* (1/10 patients) were found (Table 1). A summary of blood culture results for patients with and without IBD is presented in Table 1. From non-IBD patients *Staphylococcus* MRCNS was found in most of the positive test results (3.9% (33/844)). *Staphylococcus epidermidis* was also isolated in 9 samples (9/844). Four strains of *Staphylococcus aureus* (including 2 samples of *Staphylococcus aureus* MRSA—methicillin resistant), and *Escherichia coli* in 7 samples, including the ESBL strain producing beta-lactamases with expanded substrate spectrum in five cases, and other microorganisms (Table 1).

**Table 1:** Results of blood culture testing of IBD and non-IBD patients (January 2017–December 2019)

Number of blood cultures performed in IBD patients n	Positive results n/%	Microorganisms cultured	Number	%In relation to all samples taken	%In relation to positive results
Results of blood culture testing of IBD patients					
24	10/41.7%	*Staphylococcus epidermidis* MRCNS	4	16.7%	40.0%
		*Staphylococcus aureus*	1	4.2%	10.0%
		*Escherichia coli*	3	12.5%	30.0%
		*Cutibacterium acnes*	1	4.2%	10.0%
		*Micrococcus luteus*	1	4.2%	10.0%
Results of blood culture testing of non-IBD patients					
844	77/9.1%	*Staphylococcus epidermidis* MRCNS	33	3.9%	42.8%
		*Staphylococcus epidermidis*	9	1.1%	11.7%
		*Staphylococcus aureus* MRSA	2	0.24%	2.6%
		*Staphylococcus aureus*	2	0.24%	2.6%
		*Enterococcus faecium*	4	0.47%	5.2%
		*Enterococcus faecalis*	2	0.24%	2.6%
		*Escherichia coli*	5	0.6%	6.5%
		*Escherichia coli* ESBL	2	0.24%	2.6%
		*Enterobacter cloaceae*	4	0.47%	5.2%
		*Enterobacter aerogenes*	3	0.35%	3.9%
		*Acinetobacter baumanii*	2	0.24%	2.6%
		*Citrobacter freundii*	2	0.24%	2.6%
		*Streptococcus anginosus*	2	0.24%	2.6%
		*Pseudomonas aeruginosa*	1	0.12%	1.3%
		*Cutibacterium acnes*	3	0.35%	3.9%
		*Candida albicans*	1	0.12%	1.3%

MRCNS, methicillin-resistant coagulase-negative staphylococci (strain resistant to all beta-lactam antibiotics: penicillins, penicillins with B-lactamase inhibitor, cephalosporins and carbapenems)

MRSA, methicillin-resistant *Staphylococcus aureus* (resistant to all beta-lactam antibiotics: penicillins with inhibitors, cephalosporins, monobactams, carbapenems, except for ceftaroline)

ESBL, train with Extended Spectrum Beta-Lactamase

In a similar nine-year study conducted in Israel between 2008 and 2016, positive blood cultures in IBD patients were found in 1.3% (73/5,522) of an entire cohort of hospitalized patients. *Escherichia coli* (19/73, 26%) was the most commonly isolated pathogen causing bacteremia, including the ESBL strain producing beta-lactamases with expanded substrate spectrum in five cases (5/73, 6.85%). In addition bacteremia was also found in this cohort study caused by, among others, *Pseudomonas aeruginosa* (10/73, 14%), methicillin-sensitive *Staphylococcus aureus* (MSSA) (8/73, 11%), and methicillin-resistant *Staphylococcus aureus* (MRSA) (2/73, 3%), *Klebsiella pneumoniae* (7/73, 10%, including *Klebsiella pneumoniae* (ESBL) 2 cases representing 2.7% of all positive results), and coagulase-negative *Staphylococcus* (7/73, 10%) [7]. In a ten-year Danish study conducted from May 1999 to December 2008, it was found that among hospitalized patients the overall rate of bacteremia was 14.2 cases per 1000 admissions. During the period of analysis, a decrease was observed in the isolation of *Escherichia coli*, *Staphylococcus aureus*, coagulase-negative staphylococci and *Streptococcus pneumoniae* as etiological factors from the collected material, but there was an increase in the incidence of *Pseudomonas aeruginosa* and *Enterococcus* spp. [8]. A 6-year, nationwide French study conducted from January 1, 2009 to December 31, 2014 in a cohort of over 190,000 adults with IBD confirmed 611 cases classified as sepsis. The cause was mycobacterial and bacterial infections. An age above 65 years and extended hospital stays were determined to be risk factors for bacteremia [9]. Exacerbation of symptoms is the most common indication for hospitalization among IBD patients. Moreover, patients with inflammatory bowel disease are repeatedly exposed to antibiotic therapy [10]. This may be the reason for the emergence of antibiotic-resistant bacteria. The incidence of bacteria resistant to antimicrobial agents in our analyzed cohort of patients with IBD was 40% (4/10) of all positive blood cultures. The most commonly isolated microorganism was *Staphylococcus epidermidis* (MRCNS). In the aforementioned Israeli cohort, the incidence of highly resistant bacteria was 13.7% (10/73) [7].

A strength of this research is that the results are representative of the entire region, as the hospital provides specialist care for more than 90% of IBD patients. The limitation of the presented results is the relatively low number of observations, which indicates the need for further research in this field.

In conclusion, positive blood culture results in IBD patients are more frequent than in non-IBD patients, however, bacteremia has no impact on the overall mortality rate. The presence of IBD determines the type of microorganisms present in blood cultures. Prolonged hospitalization, rather than treatment associated with inflammatory bowel disease, is associated with an increased risk of bacteremia in hospitalized patients with IBD. The spectrum of microorganisms obtained in our research represents the local distribution and reflects the epidemic situation typical for this region.

## Data Availability

The datasets during and/or analysed during the current study available from the corresponding author on reasonable request.
